# A Comparison between Two Different Remineralizing Agents against White Spot Lesions: An *In Vitro* Study

**DOI:** 10.1155/2021/6644069

**Published:** 2021-01-19

**Authors:** Hassan Alsubhi, Mohammad Gabbani, Abdulsalam Alsolami, Mohammed Alotaibi, Jameel Abuljadayel, Waleed Taju, Omair Bukhari

**Affiliations:** ^1^Umm Al Qura University, Faculty of Dentistry, Makkah 21955, Saudi Arabia; ^2^Department of Preventive Dentistry, Umm Al Qura University, Faculty of Dentistry, Makkah 21955, Saudi Arabia

## Abstract

Enamel demineralization and white-spot lesions (WSLs) around the orthodontic brackets are common clinical complications after orthodontic fixed appliance therapy. WSLs form mainly due to plaque deposition around the brackets during the orthodontic treatment period. This study was designed to compare and evaluate the efficacy of two different remineralization agents on WSLs, which are “Clinpro 5000 and Colgate Sensitive Pro-Relief”. 27 caries-free human premolar teeth were collected after extraction for orthodontic purposes. The crowns were set in acrylic resin, and the entire surfaces were coated with nail varnish apart from an area of 4 × 4 mm on the buccal surface. The surface microhardness (SMH) was measured using the Vickers microhardness testing machine at baseline, after demineralization, and after treatment. Then, the different SMH values were statistically analyzed using mixed-effects linear regression. All samples were immersed in demineralizing solution for ten days to create WSLs, and then the teeth were allocated randomly into one of the three groups: Group 1 (control group-immersed in artificial saliva), Group 2 (treated with Colgate sensitive Pro-Relief toothpaste), and Group 3 (Clinpro 5000 toothpaste). Cycles of treatment were done for 5 minutes every 12 hours for 14 days. The samples were stored in freshly prepared artificial saliva between cycles. The mixed-effects model was used to quantify the effect of different remineralization agents. All statistics were computed using STATA software (version14.1; Stata, College Station, TX). All statistical tests were two-tailed and interpreted at the 0.05 significance level. Both agents improved the surface hardness. Clinpro 5000 improved the surface hardness by 12.7 (*P* value 0.012), and Colgate Sensitive Pro-Relief improved surface hardness by 18.2 (*P* value <0.0001), However when both treatments are compared with each other, there was no statistical significance among them. When compared to the control group, both treatments “Clinpro™ 5000 and Colgate Sensitive Pro-Relief” have significantly improved enamel's SMH.

## 1. Introduction

White-spot lesions (WSLs) are described in the literature as white clouds of a chalky appearance after air-drying a smooth enamel surface [1]. This appearance occurs due to the increased porosity in the subsurface of the enamel, which formed as a result of a carious demineralization [2].

White-spot formation is an undesirable complication around orthodontic brackets during fixed orthodontic appliance therapy. It has been reported that the prevalence rate of WSLs among orthodontic patients ranged from 25% to 46% [3, 4]. Moreover, their occurrence found to be mainly related to the enamel demineralization by organic acids formed by cariogenic bacteria, which can be accumulated rapidly around the brackets [5, 6]. A recent study found out that the most elevated levels of accumulation of biofilm in patients who are treated with orthodontic fixed appliances were related to the maxillary lateral incisors and maxillary canines, especially in the gingival third and areas behind the orthodontic arch wires [7]. Furthermore, another study found that these lesions commonly occur in the middle third of the crowns of the first molars in addition to canines and lateral incisors [8]. Those lesions would increase in size within 4 weeks, if no anticariogenic agent or a strict oral hygiene protocol was used, which highlights their rapid development [4].

Orthodontic-fixed appliances make some enamel areas inaccessible to brushing, which lead to plaque accumulation; hence, enamel demineralization would rapidly progresses [9, 10].

Demineralization is a chemical process that leads to a removal of the enamel's inorganic components by the acids that are produced by the bacteria present in the dental plaque [11, 12]; this process is considered as an inescapable side-effect of orthodontic fixed appliance treatment, particularly when the patient is not maintaining optimum oral hygiene [13]. Moreover, the demineralization process is manifested firstly as WSLs, which occurs as a result of leaching the minerals from the enamel surface and considered as a beginning of a carious lesion [12].

Saliva plays a protective role against the demineralization process, which can be in the form of reduction in tooth surface demineralization rate, and enhances the remineralization [14].

The remineralization process can be achieved by replacing the minerals in a demineralized enamel or producing amorphous mineral precipitates in the interrod and intercrystal spaces [14]. Also, remineralization can happen naturally via saliva or be induced by using therapeutic agents [14].

Various types and concentrations of remineralizing agents containing fluoride, calcium, and phosphate ions were introduced in the literature [15]. These agents help to remineralize the affected sites by monitoring the surrounding microenvironment [15]. Recently, amino acid arginine (pro-Argin technology) has been introduced as sodium monofluorophosphate into toothpaste containing 8 percent arginine, insoluble calcium carbonate, and 1450 ppm fluoride, optimized for hard dental tissue remineralization [16]. Also, Clinpro™ 5000 with 1.1% sodium fluoride is another recent anticavity toothpaste that has been shown to be effective in reducing the WSLs [17]. Although these two agents have different key ingredients, both have shown promising results in rehardening the enamel surface [16–19]. Scarce information is found in the literature when those materials' components or technologies are compared to each other. Thus, the present study aims to directly compare the potential of the two previously mentioned agents in rehardening WSLs in vitro.

## 2. Materials and Methods

The samples for this in vitro study comprised of twenty-seven caries-free human premolars that were extracted for orthodontic purposes. The teeth were checked under the microscope to make sure that they are free from restorations, cracks, white-spot lesions, and/or fractures. Afterwards, they were cleaned with an ultrasonic scaler and rechecked for cracks and white-spot lesions, to be excluded if there were any. The samples were disinfected using 0.1% thymol solution for 48 hours and soaked in distilled water. The crowns were set in acrylic resin, and the surfaces were coated with a nail varnish apart from an area of 4 × 4 mm on the buccal surface, and then they were soaked again in distilled water. The surface microhardness (SMH) was measured three times, at baseline, after creating WSLs, and after surface treatments, respectively, using the Vickers microhardness testing machine at different 3 points selected on the tooth surface under a load of 50 N for 15 seconds. The methodology was modified from a previously published study [20].

For every tooth, the baseline (SMH) was measured, and then the teeth were immersed in 10 ml of the demineralizing solution for each sample at room temperature (approximately 25°C) for ten consecutive days without refreshment to create WSLs. After that, the measurement of surface hardness (SMH) was taken again. The demineralizing agent was made in Umm Al Qura Medical Sciences Chemical Laboratory with a pH of 4.5 (2.2 mM CaCl_2_, 2.2 mM NaH_2_PO_4_, 1 ppm NaF, 100 mM NaCl, 50 mM acetic acid, and 0.02% NaN_3_).

Afterwards, the teeth were allocated randomly into one of three groups (9 samples per group). All of the teeth in the three groups were soaked and preserved in a fresh artificial saliva between treatment cycles for 14 days (0.04wt% NaCl, 0.04wt% KCl, 0.09wt% CaCl_2_.2H_2_O, 0.069wt% NaH_2_PO_4_. 2H_2_, 0.008wt% MgCl_2_.6H_2_O, 0.1wt% glucose, 0.05wt% urea, 10 ml water, 7wt% PEG 6000 0.15 mg methyl-p-hydroxybenzoate, 0.01wt% ascorbic acid, with a pH set at 6.8).

Each group was subject to the following protocols of treatment; Table 1 shows the materials that have been used per group:

Group 1 (control group) was submerged in 10 ml of artificial saliva without any additional treatment, and the solution was refreshed twice daily.

Group 2: treatment with a paste “8% arginine and calcium carbonate” Colgate Sensitive Pro-Relief” for 5 minutes every 12 hours for 14 days (28 applications), and then the paste was wiped without rinsing, followed by immersing the specimens in a fresh artificial saliva solution as in Group 1.

Group 3: treatment with a paste (Clinpro™ 5000 with 1.1% Sodium fluoride) for 5 minutes every 12 hours for 14 days (28 applications), and then the paste was wiped without rinsing, followed by soaking the specimens in a fresh artificial saliva solution.

SMH reading of each sample was taken again to evaluate the remineralization potential of each surface treatment agent, and the changes were analyzed statistically using mixed-effects linear regression. The fixed effects were time, intervention group and their interaction. While for random effects, a specific ID for every tooth had been used.

## 3. Results

Statistical analysis showed that the microhardness readings of all groups have increased after 14 days of treatment protocol when compared to the one measured after demineralization. Both agents that were used in groups 2 and 3 have significantly improved surface hardness in comparison with control. Clinpro 5000 improved the surface hardness by 12.7 (*P* value 0.012) and Colgate Sensitive Pro-Relief (group 2) improved surface hardness by 18.2 (*P* value <0.0001). However, when both treatments are compared with each other, there was no statistical significance among them as shown in Table 2.

**Figure 1:**
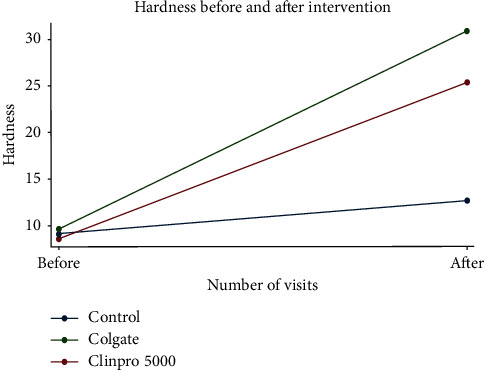
The surface microhardness before and after the treatments.

The most extreme increment in microhardness was seen in group 2 which was treated with (8% arginine and calcium carbonate; Colgate Sensitive Pro-Relief), that was followed by group 3 in descending order (Clinpro™ 5000 with 1.1% sodium fluoride) and control group as shown in Table 3 and Figure 1.

## 4. Discussion

Plaque accumulation poses a problem during fixed orthodontic appliance treatment. The different components of the appliance compromise the cleaning procedure, which make effective oral hygiene practice more difficult [9, 10]. Bacteria present in the plaque produce acids as by-products of their metabolism, which remove the inorganic materials (particularly calcium) from hard tooth surfaces. This status commonly results in the appearance of WSLs [13, 21] and is particularly of a concern if the patient is not maintaining excellent oral hygiene as it can rapidly proceed as a cavitated carious lesion [13].

Although a number of strategies have been suggested for counteraction and control of WSLs, no specific intervention has been chosen as a perfect solution for this commonplace trouble. As a result, WSLs remain a significant problem for patients and orthodontists. Given the high pervasiveness of WSLs in patients with fixed orthodontic appliances and the esthetic implications, it imposes on those patients, and the incidence of WSLs must be forestalled. Thus, the primary objective of this study was to assess the viability of two proposed therapeutic agents: “8% arginine and calcium carbonate: Colgate Sensitive Pro-Relief and Clinpro™ 5000 with 1.1% sodium fluoride” for the prevention of WSLs. The second objective was to compare the effectiveness of these two agents to find if one is more effective than the other.

Amino acid arginine (pro-Argin technology) has been introduced as sodium monofluorophosphate into the toothpaste containing 8 percent arginine, insoluble calcium carbonate, and 1450 ppm fluoride, optimized for remineralization [16]. Also, previous studies have reported that it was highly successful in reducing dentine hypersensitivity through the occlusion of dentin tubules [22–25]. This product demonstrates its remineralizing effect depending on arginine and calcium as the key components. Following the concept of organic to inorganic interactions as in tissue mineralization, the positively charged arginine acts as an organic nucleation center for mineralization [26].

Clinpro™ 5000 with 1.1% sodium fluoride is another toothpaste with proposed anticariogenic potential, that is currently accessible and has been shown to be helpful in decreasing WSLs in an underlying case report [17]. Clinpro™ 5000 toothpaste contains a component called tricalcium phosphate, while the production process is taking place; a barrier of protection is produced around the calcium that allows it to exist together with the ions of fluoride [17]. When the saliva comes in contact with the toothpaste during brushing, the barrier breaks down and makes the calcium, phosphate, and fluoride promptly accessible to the tooth surface. Both elements are normally absorbed by the tooth, which helps avoiding the onset and continuation of demineralization and promotes remineralization [17]. Amaechi (2012) [19] has shown a slightly superior remineralization effect for Clinpro™ 5000 in comparison to PreviDent Booster 5000 against WSLs in situ. They have attributed this marginal superiority to the presence of the innovative functionalized tricalcium phosphate ingredient in combination with fluoride in the product.

The primary synthesis of demineralizing agents in vitro involves acetic acid and phosphorous or lactic acid and calcium, with predetermined concentrations and ratios to have the desired pH. Furthermore, immersing the samples in this solution for a predetermined duration is a factor for creating the WSLs. Based on previous researches [27, 28], pH ranged from 3.5 to 5 of the demineralizing solutions and the period of submersion in the solution varied between two hours and 21 days which were shown to be suitable for creating WSLs on a hard dental surface. In the present study, the samples were submerged in a demineralizing solution with a pH of 4.4 for 10 days, and then the demineralization was confirmed by re-evaluating the surface microhardness (SMH) of the sample's surface.

The use of Vickers microhardness tester is one of the most commonly used and reliable methods to evaluate the SMH changes [29]. The SMH values were measured in this study before treatment at baseline, after demineralization to be confirmed, and after remineralization of samples to be detected. Both agents were found to be improving the SMH, which shows a sort of evidence for being effective as remineralizing agents not only against WSLs but suggests also their utilization in case of molar incisor hypomineralization, which is a potential area for further investigations.

Clinpro 5000 was found to improve the surface hardness by 12.7 (*P* value 0.012) and Colgate Sensitive Pro-Relief dentifrice improved the surface hardness by 18.2 (*P*-value <0.0001). However, when both treatments are compared with each other, there was no statistical significance difference among them. It is worth mentioning that the most extreme increment in microhardness was seen in group 2 which was treated with (8% arginine and calcium carbonate; Colgate Sensitive Pro-Relief), followed by group 3 (Clinpro™ 5000 with 1.1% Sodium fluoride) and then the control group in a descending order. Despite the fact that Clinpro™ 5000 contains higher fluoride parts per million (5000 ppm), a marginal better hardness results were achieved by Colgate Sensitive Pro-Relief tooth paste which contains 1450 ppm (Figure 1). This could be attributed to the difference in the key composition between materials, the active ingredients, and the mechanism of action of each treatment, which indicates a slight better hardness results with the arginine and calcium carbonate technology over the functionalized tricalcium phosphate in vitro.

## 5. Conclusion

When compared to the control group, both treatments “Clinpro™ 5000 with 1.1% Sodium fluoride and Colgate Sensitive Pro-Relief with 8% arginine and calcium carbonate (pro-Argin technology)” have improved the SMH in vitro. Although pro-Argin technology has shown higher SMH values, when both treatments are compared with each other, there was no statistical significance among them.

## Figures and Tables

**Table 1:** The composition of different materials.

Group and product	Material composition	Lot number	Manufacturer
Control (group 1)	Artificial saliva	—	Umm Al Qura Medical Sciences Chemical Laboratory

Colgate (group 2)	Toothpaste: Colgate Sensitive Pro-Relief Toothpaste containing 8.0% arginine, calcium carbonate, and 1450 ppm fluoride (monofluorophosphate)	12115	Colgate-Palmolive Swidnica, Poland

Clinpro 5000 (group 3)	Toothpaste: water-based sodium fluoride dentifrice (5,000 ppm fluoride) containing an innovative tricalcium phosphate ingredient	258100	3M ESPE dental products, USA

**Table 2:** Predicting Hardness by type of intervention via mixed-effects model.

The comparison after application of intervention	Control vs. Clinpro 5000	Control vs. Colgate	Colgate vs. Clinpro 5000
Change in hardness	12.7	18.2	5.6
*P* value	0.012	<0.0001	0.268
C. I. 95%	(2.8 to 22.5)	(8.4 to 28.1)	(−4.3 to 15.4)

**Table 3:** Mean SMH of the samples per group at baseline, after demineralization, and after treatment.

Materials	Baseline (mean ± SD)	Demineralization (mean ± SD)	Remineralization (mean ± SD)
Control (group 1)	102.4 SD ± 23.3	9.111 SD ± 10.6	12.6 SD ± 4.7
Colgate (group 2)	99.6 SD ± 28.7	9.666 SD ± 13.5	30.8 SD ± 15.4
Clinpro 5000 (group 3)	119 SD ± 31.09	8.555 SD ± 8.6	25.3 SD ± 7.2

## Data Availability

The research data used to support the findings of this study are included within the article.
